# An Individual Patient Data Meta-Analysis on Characteristics and Outcome of Patients with Papillary Glioneuronal Tumor, Rosette Glioneuronal Tumor with Neuropil-Like Islands and Rosette Forming Glioneuronal Tumor of the Fourth Ventricle

**DOI:** 10.1371/journal.pone.0101211

**Published:** 2014-07-03

**Authors:** Annika Schlamann, André O. von Bueren, Christian Hagel, Isabella Zwiener, Clemens Seidel, Rolf-Dieter Kortmann, Klaus Müller

**Affiliations:** 1 Department for Radiation Oncology, University of Leipzig Medical Center, Leipzig, Saxony, Germany; 2 Department of Pediatrics and Adolescent Medicine, Division of Pediatric Hematology and Oncology, University of Göttingen Medical Center, Göttingen, Lower Saxony, Germany; 3 Department of Neuropathology, University of Hamburg Eppendorf Medical Center, Hamburg, Germany; 4 Institute for Medical Biostatistics, Epidemiology and Informatics, University of Mainz Medical Center, Mainz, Rhineland-Palatinate, Germany; Cardiff University, United Kingdom

## Abstract

**Background and Purpose:**

In 2007, the WHO classification of brain tumors was extended by three new entities of glioneuronal tumors: papillary glioneuronal tumor (PGNT), rosette-forming glioneuronal tumor of the fourth ventricle (RGNT) and glioneuronal tumor with neuropil-like islands (GNTNI). Focusing on clinical characteristics and outcome, the authors performed a comprehensive individual patient data (IPD) meta-analysis of the cases reported in literature until December 2012.

**Methods:**

PubMed, Embase and Web of Science were searched for peer-reviewed articles reporting on PGNT, RGNT, and GNTNI using predefined keywords.

**Results:**

95 publications reported on 182 patients (PGNT, 71; GNTNI, 26; RGNT, 85). Median age at diagnosis was 23 years (range 4–75) for PGNT, 27 years (range 6–79) for RGNT, and 40 years (range 2–65) for GNTNI. Ninety-seven percent of PGNT and 69% of GNTNI were located in the supratentorial region, 23% of GNTNI were in the spinal cord, and 80% of RGNT were localized in the posterior fossa. Complete resection was reported in 52 PGNT (73%), 36 RGNT (42%), and 7 GNTNI (27%) patients. Eight PGNT, 3 RGNT, and 12 GNTNI patients were treated with chemo- and/or radiotherapy as the primary postoperative treatment. Follow-up data were available for 132 cases. After a median follow-up time of 1.5 years (range 0.2–25) across all patients, 1.5-year progression-free survival rates were 52±12% for GNTNI, 86±5% for PGNT, and 100% for RGNT. The 1.5-year overall-survival were 95±5%, 98±2%, and 100%, respectively.

**Conclusions:**

The clinical understanding of the three new entities of glioneuronal tumors, PGNT, RGNT and GNTNI, is currently emerging. The present meta-analysis will hopefully contribute to a delineation of their diagnostic, therapeutic, and prognostic profiles. However, the available data do not provide a solid basis to define the optimum treatment approach. Hence, a central register should be established.

## Background

In the most recent update of the World Health Organization (WHO) classification of central nervous system (CNS) tumors [29], [30], three new entities have been added to the repertoire of glioneuronal tumors: papillary glioneuronal tumor (PGNT) (WHO grade I), rosette-forming glioneuronal tumor of the fourth ventricle (RGNT) (WHO grade I), and rosetted glioneuronal tumor with neuropil-like islands (GNTNI) (WHO grade II/III) [29], [40]. PGNT, a mixed tumor consisting of glial und neuronal histological differentiation, shows a typical structure of GFAP-positive psedopapillae surrounded by an interpapillary (neuronal) zone [2]. Necroses and elevated mitotic activities are rarely seen [3]. The biphasic histopathology, consisting of neurocytic and glial architecture, is also typical for RGNT [29], [30]. Neurocytes of the neuronal component shape rosettes with eosinophilic, synaptophysin-positive cores and perivascular pseudorosettes. The glial part of the tumor, showing similar features like pilocytic astrocytoma, represents the larger portion [39]. In contrast, GNTNI shows features of a high-grade glioma, mostly interpreted as astrocytic [43], but ependymal or oligodendroglial differentiation is possible [12]. Dispersed in this glial component, the most prominent feature of these tumors, rosetted neuropil-like islands, can be found [37].

Although morphological, immunohistochemical, and molecular features have been intensively investigated over recent years [29], [30], clinical features, current treatment approaches, and prognosis are still elusive. The pertinent literature on the topic is primarily limited to single- case reports or small case series and do not provide a comprehensive overview. In 2009, Allende et al. aimed to summarize the pathological and clinical findings of PGNT, RGNT, and GNTNI [3]. However, a major methodological shortcoming of their review is based on the fact that a systematic literature search was not performed. Accordingly, their findings may be biased by the authors’ personal opinions or the selection of publications included in their analysis. In particular, the variety of articles published during the past four years may contribute valuable new information toward the understanding of the three previously mentioned entities. The purpose of this individual patient data meta-analysis was to increase the current knowledge about clinical features, treatment, and outcome of PGNT, RGNT and GNTNI.

## Materials and Methods

### Scientific question

The purpose of the present IPD meta-analysis was to assess the clinical characteristics and outcome of the patients with PGNT, RGNT and GNTNI reported in the literature.

### Search strategy and selection criteria

**Figure 1:**
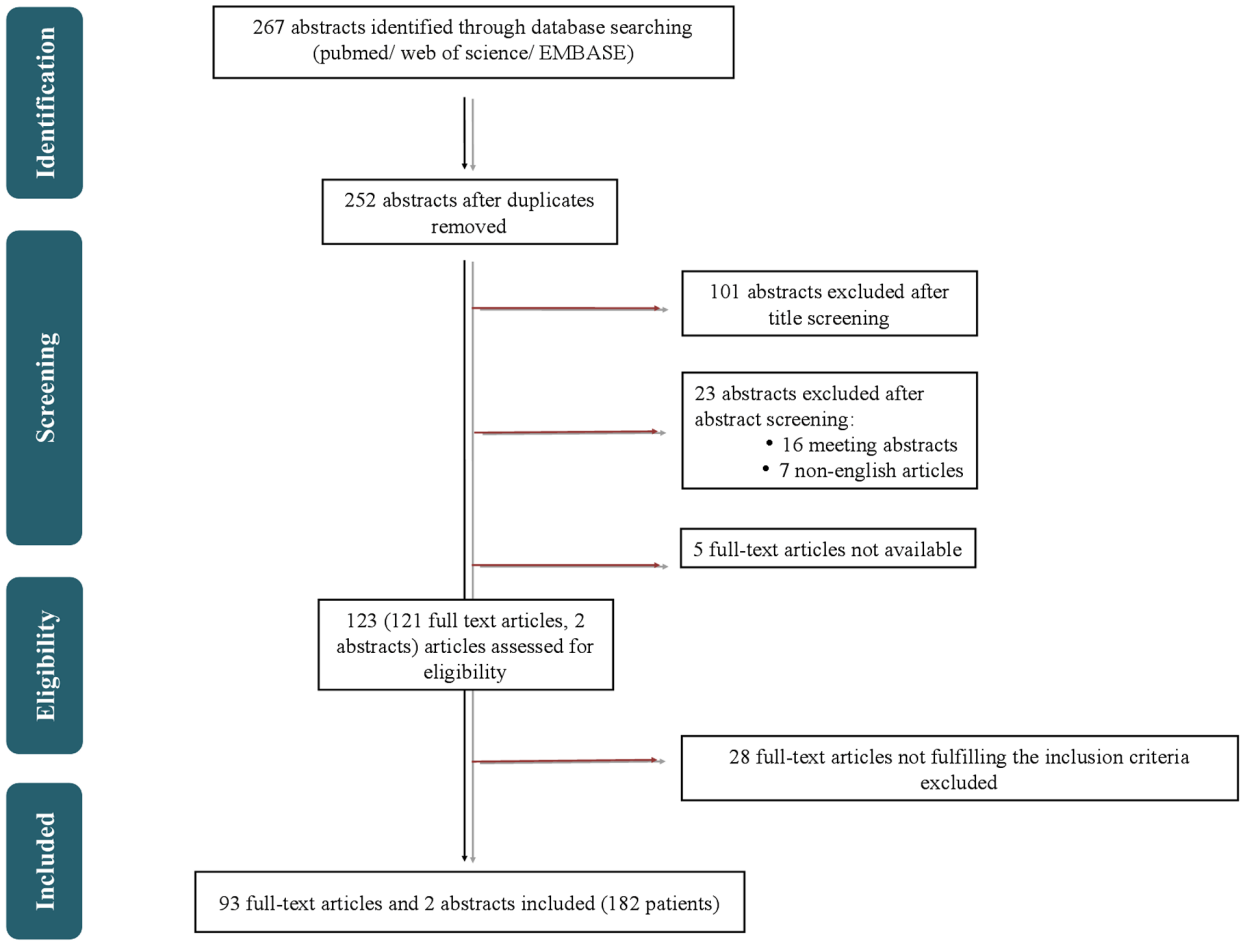
Systematic literature search. Procedure of publication retrieval and in- and exclusion of cases is displayed in a PRISMA (preferred reporting items for systematic reviews and meta-analyses) flow chart.

The authors searched PubMed, Embase and Web of Science from January 1998 to December 2012 (the last update to all databases was on December, 17, 2012) for published articles with predefined search terms without language restrictions. The search was assisted by an experienced librarian (Mrs. Christiane Hofmann; library of the University of Leipzig). The keywords were (1) (papillary) AND (glioneuronal OR glioneural) AND (tumor OR tumour OR neoplasm), (2) (rosette forming OR rosetted) AND (glioneuronal OR glioneural) AND (tumor OR tumour OR neoplasm), (3) neuronal AND (glioneuronal OR glioneural) AND (tumor OR tumour OR neoplasm); (1) OR (2) OR (3). The process of publication retrieval and in- and exclusion of cases is displayed in a PRISMA (preferred reporting itmens for systematic review and meta-analysis) flow chart [34] (Fig. 1).

The authors identified 267 hits from a search of PubMed, Embase, and Web of Science databases. These were imported in reference management software (endnote.com X6.0.1). Titles and abstracts were reviewed by one researcher (AS). Duplications were excluded (n  = 15). In addition to the obvious duplicates (e.g. same articles twice), we identified identical cases by reviewing investigators in the study and patient characteristics. Results obtained on the same cohort of cases in multiple publications were collected only once, similar as described in a previos study [32], by including the largest series of patients.

Furthermore the authors excluded 101 abstracts because the subjects were not related to the aforementioned search terms. Case- series or cohort studies reporting on papillary glioneuronal tumor (PGNT) (WHO grade I), rosette-forming glioneuronal tumor of the fourth ventricle (RGNT) (WHO grade I), and rosetted glioneuronal tumor with neuropil-like islands (GNTNI) (WHO grade II/III) were included.

Another 16 meeting abstracts were excluded as well as five abstracts with insufficient data for which no full-text was available despite interlibrary loan. One exception was made, including one abstract providing sufficient information about one case without the available full-text.

Nine non-English articles in Polish, Chinese, Russian, Japanese and Slovakian were found. For eight of these nine articles, no full-texts were available; however there was one article with an English abstract and available Chinese full-text. For inclusion, the authors translated this article and contacted the authors of the study to receive sufficient data for the meta-analysis. For three articles the English abstracts were disposable, of which two were reviews and did not include original data. Those two reviews were therefore excluded. The remaining abstract (case report) was included, despite limited data. To complete the limited data we contacted the authors of the study, but no responses were received in time. The remaining five articles were excluded because neither the full-texts nor the abstract were available (Table 1).

**Table 1:** Limitation of this study - non-English articles.

	Language	Case report(y/n)	Numberof cases^a^	Abstract y/n	Languageof abstract	Information delivered
1	Polish	n/s	n/s	**no**	n/s	–
2	Chinese	yes	1	**no**	n/s	–
3	Russian	yes	1	**yes**	English	histology, age+gender of patient, tumor location, **no symptoms/treatment/PFS/OS**
4	Chinese	yes	1	**no**	n/s	–
5	Chinese	yes	1	**yes**	English	age+gender of patient, symptoms, MRI-character (solid/cystic), tumor location, histology, treatment, follow-up
6	Chinese	yes	2	**no**	n/s	–
7	Chinese	yes	2	**no**	n/s	–
8	Japanese	no	0	**yes**	English	WHO classification 2007
9	Slovakian	no	0	**yes**	English	WHO classification 2007

Note: n/s – not specified; y/n – yes/no.

a Provided by title of the article or abstract if available.

Overall, the authors assessed 123 eligible articles, 121 full-texts, and 2 abstracts. Possible additional studies were traced by checking the reference lists of selected publications, but they did not provide any further articles. Papers were reviewed by two authors (AS and AOvB). Disagreements were resolved through discussion and consensus with a third author (CH and/or KM). In case of uncertainty with regard to histopathological diagnosis, CH assessed whether diagnoses were based on mandatory analysis for the 2007 WHO classification of tumors of the central nervous system: Data from five patients whose tumor could not be histologically categorized according to the 2007 WHO grading system were excluded (part of the 28 excluded full-texts). The following criteria were used: (1) published immunohistochemistry, (2) growth pattern as described by WHO 2007 classification, and (3) case report considered as typical example by authors.

### Data collection, quality control, and data synthesis

Information on the symptoms at diagnosis, histopathological diagnoses, patient characteristics, MRI findings, treatments, and outcomes were recorded on a standard data extraction form.

To ensure correct histopathological diagnosis according to the criteria defined by the 2007 WHO classification of tumors of the central nervous system, articles published before 2007 were included only if they were cited in the 2007 WHO classification of tumors of the central nervous system (“blue book”) or after a detailed assessment of the description of the analysis was performed to diagnose a case by an experienced neuropathologist (CH) in order to check whether criteria to diagnose a case according to the 2007 WHO classification of tumors of the central nervous system are fulfilled.

Any obvious errors (plausibility tests), inconsistencies with publication, inconsistencies between variables, or extreme values were discussed with the authors (CH and KM) and corrected where necessary.

### Statistics

Study- level data were collected (95 studies; 182 patients). Because of the small number of patients per study, the study-level characteristics are not presented for each individual case report or case series. A one-stage approach according to Simmonds et al. was used to pool all data into a single master database [44].

PFS was defined, as described elsewhere [18], as the time from date of diagnosis to first progression or relapse or tumor-related death. Last contact (without an observed event) or death unrelated to progression or relapse required censoring.

For overall survival (OS) death by any cause was taken into account. Survival times were calculated from the date of diagnosis onwards. The Kaplan–Meier method was used to estimate PFS and OS rates. PFS estimates were compared by means of the log rank test. In some cases the date of diagnosis differed from the date of surgery. Therefore, the influence of the extent of resection on the PFS was not assessed. In addition to the assessment by means of the Kaplan-Meier method and the log rank test, each variable was tested individually in a Cox proportional hazards model using the change in log likelihood from the null model. All analyses are exploratory; therefore, no significance level was fixed. All analyses were performed using SPSS, version 20 (SPSS Inc., Chicago, IL, USA).

### Ethical standards

This manuscript is in accordance with the ethical standards established in the 1964 Declaration of Helsinki and its subsequent amendments.

## Results

**Figure 2:**
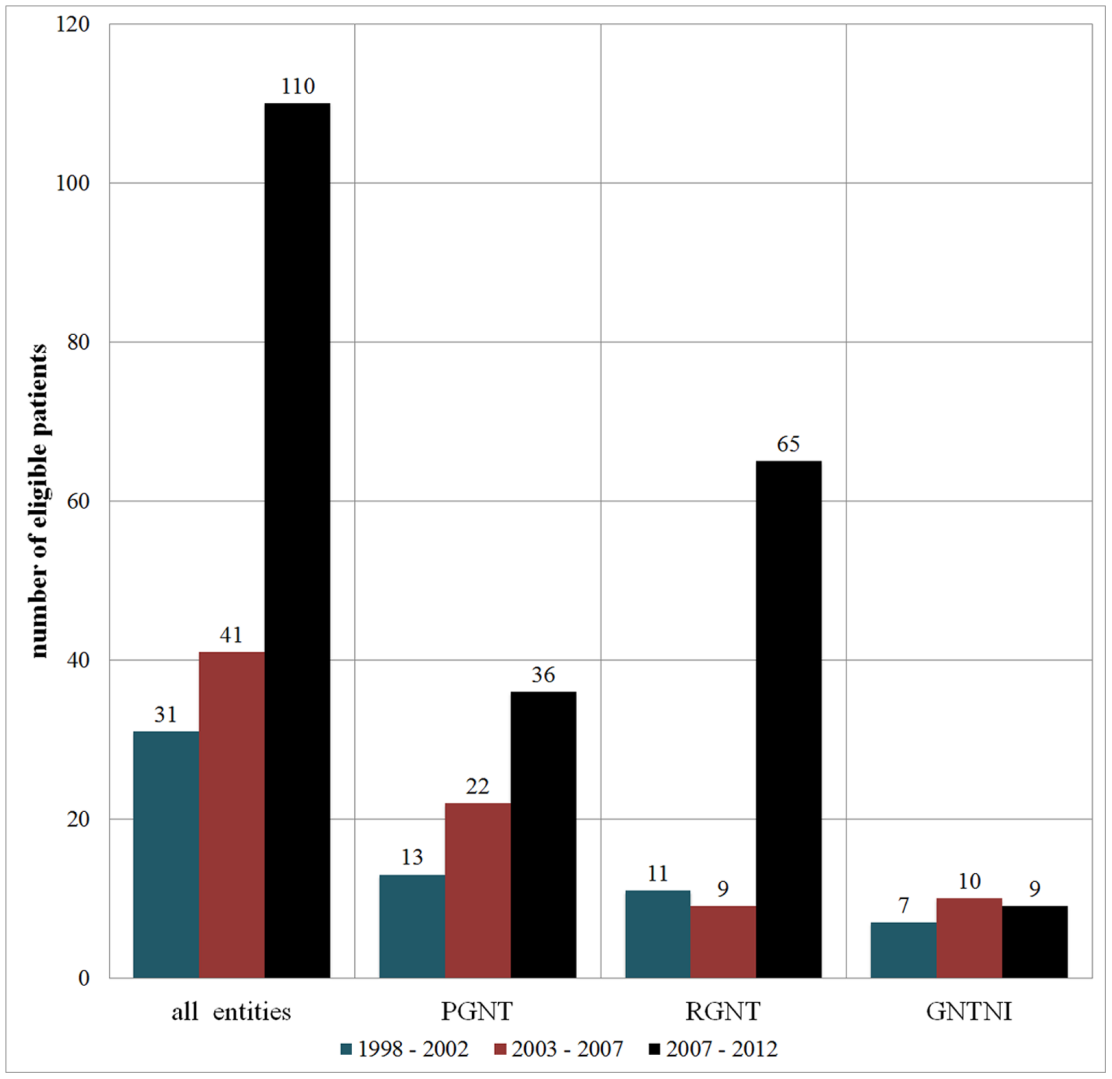
Number of published case reports. There is an increasing number of case reports over the last years.

The authors evaluated the full text articles of 95 publications reporting on 182 patients with PGNT (n  = 71), RGNT (n  = 85), and GNTNI (n  = 26) (Fig. 2). A detailed description of references containing patient data is provided in File S1.

### Clinical characteristics and first-line treatment

#### PGNT

**Figure 3:**
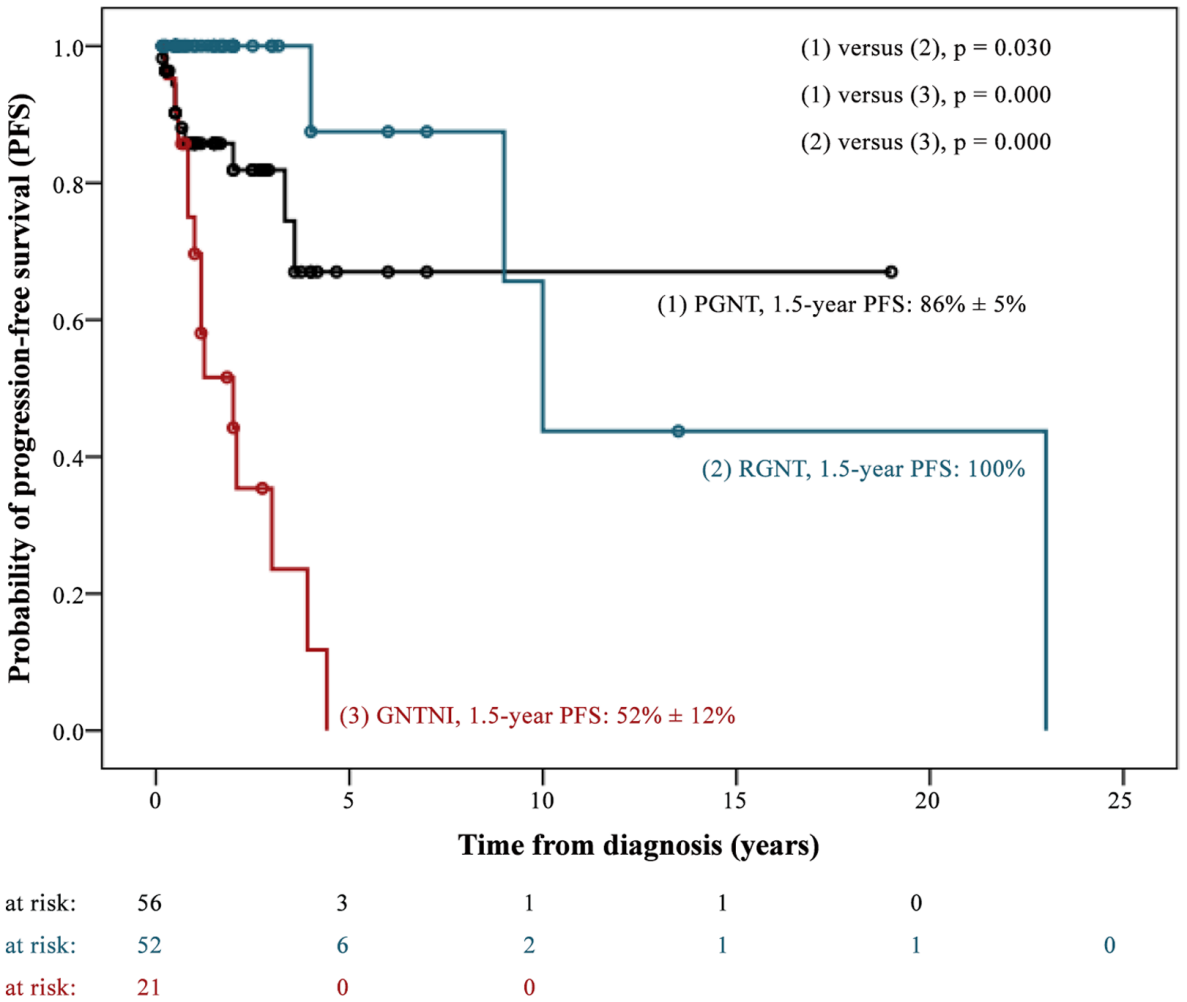
Kaplan-Meier Plot PFS.

**Figure 4:**
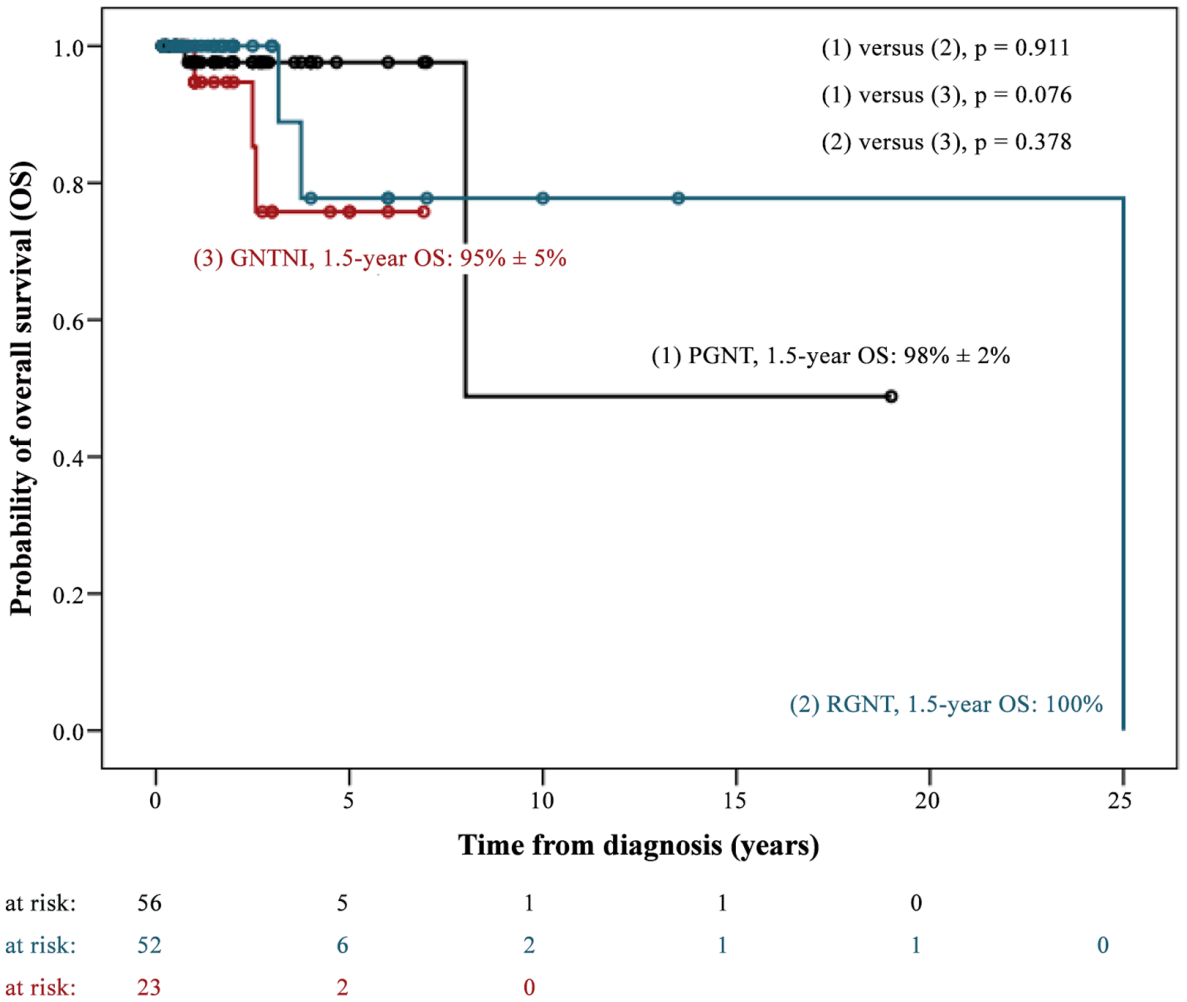
Kaplan-Meier Plot OS.

In total, we assessed 71 patients with PGNT. Thirty- three (46.5%) were male. Median age at diagnosis was 23 years (range, 4–75 years). The majority of PGNT (97.2%) were located in the supratentorial region. Primary metastatic dissemination was evident in one case (Table 2). In 64 out of 71 patients (90.1%) diagnosis was preceded by neurological symptoms, most frequently headache, seizures, and nausea/vomiting (Table 3). Complete resection was reported in 52 cases. Two patients received adjuvant radiation therapy after complete tumor resection (1x focal RT with 55 Gy, 1x no RT details available). Six patients with incomplete tumor resection underwent adjuvant radiotherapy (n  = 2), chemotherapy (n  = 1), or both (n  = 3). RT details were not reported except in one case (focal RT with 45 Gy). The outcome was reported in 57 out of 71 patients. Ten patients progressed and two patients died. The median follow-up time of surviving patients was 1.5 years (0.2–19.0 years) (Table 2). The 1.5-year PFS and OS rates were 86% ±5% and 98% ±2% (2-year PFS were 82% ±6% and OS 98% ±2%, respectively) (Figs. 3 and 4).

**Table 2:** Clinical Characteristics of ….

	PGNT (n = 71)		RGNT (n = 85)		GNTNI (n = 26)	
Characteristics	n	%	n	%	n	%
**Gender**						
Male	33/71	46.5	39/85	45.9	17/26	65.4
Female	38/71	53.5	46/85	54.1	9/26	34.6
**Age at diagnosis**						
Median (range)	23.0 years (4.0–75.0 years)		27.0 years (6.0–79.0 years)		40.0 years (2.0–65.0 years)	
Children (<18 years)	25/71	35.2	18/85	21.2	6/26	23.1
Adults (≥18 years)	46/71	64.8	67/85	78.8	20/26	76.9
Patients <26 years	42/71	59.2	40/85	47.1	8/26	30.8
Patients ≥26 years	29/71	40.8	45/85	52.9	18/26	69.2
**Tumor location**						
supratentorial	69/71	97.2	13/85	15.3	18/26	69.2
Posterior fossa	1/71	1.4	68/85	80.0	–	–
Spinal	–	–	1/85	1.2	6/26	23.1
More than one area	1/71	1.4	3/85	3.5	2/26	7.7
**Primary metastasis**	1/71	1.4	6/85	7.1	4/26	15.4
**Proliferation index (Ki-67)**						
Ki-67 reported	55/71	77.5	64/85	75.3	24/26	92.3
Median (range) (%)	1.8 (0.5–50.0)		1.0 (0.4–4.9)		4.0 (1.0–20.0)	
>1.6%	27/55	49.1	18/64	28.1	21/24	87.5
≤1.6%	28/55	50.9	46/64	71.9	3/24	12.5
**Tumor size (cm), reported**	51/71	71.8	36/85	42.4	5/26	19.2
median (range)	4.0 (1.0–9.0)		3.0 (0.5–9.6)		4.5 (3.5–6.0)	
>3.5 cm	30/51	58.8	8/36	22.2	3/5	60.0
≤3.5 cm	21/51	41.2	28/36	77.8	2/5	40.0
**Character in imaging, reported**	61/71	85.9	51/85	60.0	26/26	100.0
solid	5/61	8.2	19/51	37.3	19/26	73.1
cystic parts	56/59	91.8	32/51	62.7	7/26	26.9
**Extent of tumor resection**						
Complete resection	52/65	80.0	36/63	57.1	7/25	28.0
Incomplete resection	13/65	20.0	27/63	42.9	18/25	72.0
Not reported	6/71	8.5	22/85	25.9	1/26	3.8
**Follow-up time of survivors**						
Median (range)	1.5 years (0.2–19.0 years)		1.2 years (0.2–13.5 years)		1.7 years (0.6–7.0 years)	
**Outcome**						
Assessable for PFS	57/71	80.3	52/85	61.2	21/26	80.8
Disease progressions	10/57	17.5	4/52	7.7	14/21	66.7
Assessable for OS	57/71	80.3	52/85	61.2	23/26	88.5
Deaths	2/57	3.5	3/52	5.8	3/23	13.0

**Table 3:** Initial symptoms and radiology features.

	PGNT (n = 71)		RGNT (n = 85)		GNTNI (n = 26)	
Characteristics	n	%	n	%	n	%
**Information about initial symptoms provided**	66/71	93.0	66/85	77.6	26/26	100.0
**symptomatic**	64/66	97.0	58/66	87.9	26/26	100.0
headache	39/64	60.9	43/58	74.1	3/26	11.5
nausea/vomiting	15/64	23.4	15/58	25.9	2/26	7.7
abnormality of gait and coordination	3/64	4.7	18/58	31.0	2/26	7.7
papilloedema or optic atrophy	8/64	12.5	6/58	10.3	0/26	0.0
seizures	21/64	32.8	2/58	3.4	17/26	65.4
visual disturbance	12/64	18.8	9/58	15.5	0/26	0.0
**Radiology**						
**Character in imaging, reported**	61/71	85.9	51/85	60.0	26/26	100.0
solid	5/71	7.0	19/51	37.3	19/26	73.1
cystic	8/61	13.1	11/51	21.6	0/26	0.0
cystic with mural nodule	23/61	37.7	0/51	0.0	5/26	19.2
cystic and solid	25/61	41.0	21/51	41.2	2/26	7.7
**enhancement, reported**	63/71	88.7	61/85	71.8	26/26	100.0
enhancement	60/63	95.2	44/61	72.1	14/26	53.8
**density in MR-imaging T1**	44/71	62.0	52/85	61.2	12/26	46.2
hypointens	41/44	93.2	48/52	92.3	11/12	91.7
hyperintens	3/44	6.8	–	–	1/12	8.3
isointens	–	–	4/52	7.7	–	–
**density in MR-imaging T2**	39/71	54.9	47/85	55.3	12/26	46.2
hypointens	0/39	0.0	–	–	1/12	8.3
hyperintens	39/39	100.0	40/47	85.1	11/12	91.7
isointens	–	–	7/47	14.9	–	–

#### RGNT

The authors evaluated 85 patients [men, 39 (45.9%)] with RGNT. The median age at diagnosis was 27 years (range, 6–79 years). Eighty percent of the tumors were located in the infratentorial region. Six patients presented with primary metastatic spread (Table 2). In 58 out of 85 cases (68.2%) the diagnosis was preceded by neurological symptoms, most frequently headache, abnormalities of gait and coordination, and nausea and vomiting (Table 3). Complete resection was achieved in at least 36 cases. Three patients with incomplete tumor resection received focal RT (total doses 46, 55, and 57 Gy, respectively). The outcome was reported in 52 out of 85 patients (61.2%). Four patients progressed and three patients died. The median follow-up time of surviving patients was 1.2 years (0.2–13.5 years) (Table 2). The PFS and OS rates at 1.5 and 2 years after diagnosis were 100%, respectively (Figs. 3 and 4).

#### GNTNI

Twenty-six patients with GNTNI were investigated. Seventeen (65.4%) were male. The median age at diagnosis was 40 years (range, 2–65 years).

Tumors were in the supratentorial (69.2%) and spinal (23.1%) regions. Four patients showed initial metastases (Table 2). All patients were symptomatic when diagnosed with seizures and headache being the most frequent clinical signs (Table 3). Gross total resection was not achievable in 18 out of 26 patients. Ten with incomplete (n  = 9) or unknown extent of tumor resection (n  = 1) underwent adjuvant focal radiotherapy (n  = 4), irradiation of the craniospinal axis (n  = 1, disseminated disease), or focal radiotherapy and chemotherapy (n  = 5). The maximum RT doses were reported in five cases (2 × 60 Gy, 50 Gy, 59.4 Gy, and 50.4 Gy). Two patients underwent adjuvant treatment (chemotherapy, n  = 1; RT and chemotherapy, n  = 1) despite complete tumor resection. The outcome was reported in 21 patients. Fourteen patients progressed and three patients died. The median follow-up time of surviving patients was 1.7 years (0.6–7.0 years) (Table 2). Progression-free and overall survival rates at 1.5 years after diagnosis were 52% ±12% and 95% ±5%, respectively (Figs. 3 and 4). Two-year PFS was 44% ±12% and OS 95% ±5%.

### Evaluation of potential prognostic factors for PFS across all three entities

#### Univariable analyses, Kaplan-Meier method and log rank test

Neither gender (p  = 0.315) nor age (cut-off of 18 years; p  = 0.846; cut-off of 26 years as median age for all patients: p  = 0.575) had an on PFS. In contrast, univariable survival analyses identified histology (p<0.001), WHO grading (p<0.001), the Ki-67 proliferation index (cut-offs of 1.6 and 5.0%, respectively) (1.6%, p  = 0.002; 5.0%, p<0.001), the maximum tumor diameter as measured on imaging [cut-off 3.5 cm (median size of tumor) (p  = 0.028)], and the occurrence of cystic tumor parts (p  = 0.015) as critical factors for PFS. Patients with primary metastatic disease tended to progress earlier (p  = 0.054) (Fig. 3, Table 4).

**Table 4:** Impact of potential prognostic factors on progression.

Factor	n =	2-year PFS (%)	1.5-year PFS (%)	p =
**Histology**				
Assessable for PFS	130/180 (71.4%)			
PGNT	57/130 (43.8%)	82±6	86±5	**0.000**
RGNT	52/130 (40.0%)	100	100	
GNTNI	21/129 (16.2%)	44±12	52±12	
**WHO Grade**				
Assessable for PFS	130/182 (71.4%)			
WHO °I	109/130 (83.8%)	90±4	93±3	**0.000**
WHO ° II/III	21/130 (16.2%)	44±12	52±12	
**Gender**				
Assessable for PFS	130/182 (71.4%)			
Male	62/130 (47.7%)	79±7	86±5	0.315
Female	68/130 (52.3%)	82±5	82±5	
**Age**				
Accessible for PFS	130/182 (71.4%)			
Children (<18 years)	39/130 (30.0%)	77±8	83±7	0.846
Adults (≥18 years)	91/130 (70,0%)	82±5	85±4	
Patients <26 years	68/130 (52.3%)	83±6	86±5	0.575
Patients ≥26 years	62/130 (47.7%)	78±7	82±6	
**Primary metastasis**				
Accessible for PFS	130/182 (71.4%)			
yes	9/130 (6.9%)	51±20	51±20	**0.054**
no	121/130 (93.1%)	83±4	87±4	
**Proliferation index (Ki-67) (%)**				
Accessible for PFS	111/182 (61.0%)			
>1.6	50/111 (45.0%)	92±5	92±5	**0.002**
≤1.6	61/111 (55.0%)	66±8	73±7	
≥5	93/111 (83.8%)	44±13	53±12	**0.000**
<5	18/111 (16.2%)	88±5	90±4	
**Maximum tumor diameter (cm)**				
Accessible for PFS	73/182 (40.1%)			
>3.5	36/73 (49.3%)	81±7	81±7	**0.028**
≤3.5	37/73 (50.7%)	97±3	97±3	
**Appearance on imaging**	137/182 (75.3%)			
Accessible for PFS	113/182 (62.1%)			
Exclusively solid	31/113 (27.4%)	68±11	76±9	**0.015**
cystic parts	82/113 (72.6%)	85±5	87±4	

#### Cox proportional hazards regression analysis (continuous variables)

In the univariable cox regression analysis age (p  = 0.128, hazard ratio  = 1.02 per year, 95% CI: 0.99–1.04) and the maximum tumor diameter (p  = 0.057, hazard ratio  = 1.338 per cm, 95% CI: 0.992–1.805) did not influence PFS, whereas the proliferation index of Ki-67 (p  = 0.003, hazard ratio  = 1.10 per %, 95% CI: 1.03–1.18) did.. As an example, a 1% increase in Ki-67 positive tumor cells extended the risk of progression by 10% and a 10% increase in Ki-67 positive tumor cells by 259% (1.1^10^). For the categorical variables (WHO grading, gender, primary metastasis and the occurrence of cystic tumor parts), the results delivered by the Kaplan-Meier method and the log rank test were confirmed (Table 5).

**Table 5:** Cox Regression.

Factor	p =	hazard ratio (HR)	95% confidence interval (HR)
**WHO Grade**			
°I			
°II/III	**0.000**	0.137	0.062–0.305
**Gender**			
Male	0.320	0.675	0.312–1.463
Female			
**Age**	0.128	1.017	0.995–1.039
**Primary metastasis**			
yes			
no	0.069	0.316	0.091–1.095
**Proliferation index (Ki-67) (%)**	**0.003**	1.102	1.033–1.175
**Maximum tumor diameter (cm)**	0.057	1.338	0.992–1.805
**Appearance on imaging**			
Exclusively solid	**0.020**	2.659	1.168–6.052
Cystic parts			

## Discussion

### General aspects

PGNT, RGNT, and GNTNI have been raising more and more awareness in (clinical) neuro-oncology over recent years. This was particularly underscored by the special recognition given to them in the latest update of the WHO classification of CNS tumors [29], [30], [40]. Meanwhile, a considerable number of case reports and small case series have been published (Fig. 2). Now, studies extracting and subsequently interpreting the available data are highly needed. Recently, Zhang et al. assessed a total of 41 RGNT patients reported in the literature between 2002 and 2012 [52]. However, comparable studies for PGNT and GNTNI do not exist so far to the best of our knowledge. The aim of the present study is to provide a comprehensive meta-analysis for all three entities. Through a systematic literature search, we were able to generate a data set containing 71 cases of PGNT, 85 cases of RGNT, and 26 cases of GNTNI.

### PGNT

PGNT is a rare tumor of the central nervous system, first described by Komori et al. in 1998 [24]. Histopathological features, including biphasic components of glial and neuronal pattern as well as radiological characteristics such as frequent occurrence of a cystic lesion with mural nodule (39%, Table 3) or mixed solid-cystic lesions (39%, Table 3) with ring-like enhancement in MRI, are able to facilitate the diagnosis [9], [50]. However, the differentiation among ganglioglioma, pleomorphic xanthoastrocytoma, pilocytic astrocytoma and dysembryoplastic neuroepithelial tumor can sometimes be challenging [7], [39], [49].

When defined as a WHO grade I tumor, a benign course and an excellent prognosis can be assumed, especially when presenting with a low proliferation index or after gross total tumor resection (GTR). This study's PGNT showed a 1.5-year PFS of 86% ±5% and a 1.5-year OS of 98% ±2%, respectively (Figs. 3 and 4). Even cases with an elevated proliferation index showed a favorable course [6], [10], [20], [22], [23], [35]. As a single exception to the rule one case of PGNT recurred despite GTR and low proliferation [23]. Therefore, no certain correlation between outcome and the extent of the tumor resection or the proliferation index can be made, which might give the impression that genetic alterations of PGNT may be a key issue for our understanding [28].

PGNT mostly occurs in young adults, but with a wide range in age (median age 23.0 years, Table 2). Initial symptoms can be seen in almost every case (64/71 patients, 90%, Table 3), resulting in physical examination and diagnostic services such as CT or MRI. At the time of diagnosis, a median tumor size of 4.0 cm (range 1.0–9.0) was shown in radiological imaging. In most cases enhancement of the tumor was reported (60/71 patients, 85%, Table 3), in addition to hyperintensity in T2-MR imaging (Table 3).

Most patients (80%, Table 2) received GTR and had an excellent prognosis, indicating that this is the first-choice treatment for PGNT [10], [11], [13], [27], [38]. Any additional therapy such as radiotherapy or chemotherapy appears to be necessary only in a minority of patients. Whether adjuvant treatment was administered for PGNT patients was reported in 31 (chemotherapy) and 35 (radiotherapy) out of 71 cases (45 and 49%, respectively). Four patients received chemotherapy (6% of all cases), all after STR, and seven patients got radiotherapy (10% of all cases; five after STR, two after GTR). However, there might be the possibility that more patients received adjuvant therapy than the authors are aware because of the low rate of reported data. Possible reasons for adjuvant treatment include high proliferation index, inoperability and progressive disease [16], [47]. Regular radiological monitoring is necessary to detect any recurrence of tumor. In this case, surgical intervention should be considered first [8].

### RGNT

First described as dysembryoplastic neuroepithelial tumor (DNT) of the cerebellum by Kuchelmeister et al. in 1995 [26], Komori et al. defined RGNT as a specific disease in 2002 [25]. RGNT is a rare tumor of the central nervous system, typically arising in the III and IV ventricles. An increasing number of patients are now known with RGNT outside the characteristic location, such as in the pineal region, optic chiasm, spinal cord and septum pellucidum [5], [19], [42], [45], [51]. Radiologically, a solid or mixed solid/cystic tumor can be found in 37 and 41%, respectively, usually enhancing (72% of 61 reported cases), with hyper-intense signals in T2-MRI (85%) and iso- or hypo- intense signals in T1-MRI (92%, Table 3) [52]. Similar to PGNT, RGNT histologically consists of both glial and neuronal components [43]. Pseudorosettes are the most characteristic feature. Against the background of a histological similarity to DNT, some publications discussed that RGNT might be the infratentorial version of cerebral DNT [25], [26]. Differential diagnoses include pilocytic astrocytoma, ependymoma, oligodendroglioma, central neurocytoma and DNT, of course [43], [51]. Thus, a distinct diagnosis might be demanding.

Occurring primarily in young adulthood (median age 27.0 years), one case of a 79-year-old patient provides some evidence that this tumor may also occur in older persons [31].

Classified as a WHO grade I tumor, RGNT is characterized by a favorable prognosis upon surgical resection: a 1.5-year PFS and OS of 100% was achieved in RGNT patients in this study’s data set (Figs. 3 and 4). However, local recurrences have been reported as well as disseminated disease in 7% (Table 2) [15], [48], leading to the hypothesis that GTR is the treatment of first choice. As far as the authors are aware, only about half of the patients received GTR, leaving many cases with subtotal resection (STR) (Table 2). Some authors even recommended performing a biopsy only [25], [48]. Zhang et al. (2013) could not show any difference in survival when comparing patients with GTR versus STR [52], which might be the result of the small number of cases.

Even though it is challenging because of the delicate tumor location, surgery seems to remain the most important first-line therapy, whereas radiotherapy should be considered as adjuvant treatment for progressive or disseminated diseases as well as definitive treatment in case of inoperability [25], [48], [52]. Data about given adjuvant treatment for RGNT was reported in 76% of this study’s cases: Three patients received radiotherapy, which is 4% of all cases (all after STR), but no chemotherapy was given (data not shown). The robustness of this information might be slightly limited because of the possibility that more patients may have received chemotherapy and/or radiotherapy.

### GNTNI

In 1999, Teo et al. described GNTNI as a scarce tumor that differs from PGNT and RGNT [46]. Characterized as a WHO grade II or III tumor, it is the only one of these three entities that is not part of the glioneuronal tumor category [3]; instead, it is categorized as astrocytoma [29], [30]. GNTNI appears with a biphasic histology consisting of neurocytic cells that surround distinctive oval neuropil-rich islands and fibrillary, protoplasmic, or gemistocytic astrocytes as part of the glial component [1], [2], [41]. This glial part exposes anaplastic features such as increased cellularity, frequent mitosis and necrosis, nuclear pleomorphism, high proliferation index, and/or microvascular proliferation. In this study’s analysis, GNTNI showed a median proliferation index of 4.0% (range 1.0–20.0%), compared with a median proliferation index of 1.6% for PGNT and 1.0% for RGNT (Table 2). This might help to differentiate this tumor from both PGNT and RGNT. Differential diagnoses include ependymomas (with neuropil-like islands), oligodendrogliomas (with neurocytic differentiation), or RGNT.

Most of these tumors are located in the supratentorial region (69%); however, spinal (23%) and even disseminated disease at primary diagnosis (8%) have been described by authors frequently (Table 2) [17], [21], [37], [41]. Showing variable contrast enhancement (Table 3), GNTNIs appear mostly as solid tumor in 73%, or, a smaller amount of 19%, cystic with a mural nodule with T2-hyperintensity (92%) and T1-hypointensity (92%) (Table 3) [4].

GNTNI shows an unfavorable prognosis when compared with RGNT and PGNT (1.5-year PFS 52% ±12%, 1.5-year OS 95% ±5%; Figs. 3 and 4), which is reflected in the histomorphology and the grading. Most of all published cases have been treated with incomplete resection (72%, Table 2). Again, this increases the risk of local recurrence. The best therapy for patients with GNTNI remains a serious challenge. Radiotherapy and chemotherapy as adjuvant treatments after resection are important and constitute a cornerstone of the treatment that should be discussed for every patient. A total of 21 out of 26 (81%) cases reported about adjuvant treatment: seven patients received chemotherapy (27% of all cases) and 11 patients radiotherapy (42% of all cases) (data not shown). Most patients received adjuvant treatment after an incomplete resection of the tumor (10/11 radiotherapy and 5/7 chemotherapy). For disseminated disease, a definitive radiotherapy or radiochemotherapy is conceivable but must be considered as an experimental approach. Even though much remains uncertain, treatment of GNTNI may include the cornerstones of the treatment of diffuse astrocytoma, because of the histopathological similarity.

### Limitations of this study

To the author’s knowledge, we generate the first analysis containing progression-free and overall-survival for these three entities. It turns out that PGNT, RGNT and GNTNI demonstrate good survival rates. However, the authors cannot fully eliminate several limitations of the study.

First, the follow-up period is too short to draw definitive conclusions. Therefore, a registry study is needed for these rare tumors to collect follow-up data over a longer period.

Second, without a doubt, the patients in this study underwent very heterogeneous treatments. This may bias outcome and prognosis.

Third, unfortunately the authors failed to find every single full-texts for every abstract or hit, especially for the non-English case studies. Thus, two of the 95 case- series or cohort studies were assessed by abstracts only, and seven non-English hits were excluded entirely, resulting in another limitation of this study.

### Statistical limitations

On univariate analyses the authors identified a variety of potential risk factors for PFS (Fig. 3, Table 3). However, it is highly improbable that all these factors are independent of each other. The simultaneous s of several variables on survival times are usually investigated by means of the Cox proportional hazard regression model. However, for the results to be reliable, the number of events (e.g. disease progressions or relapses) must be high enough. For each variable investigated, at least 10 events are required [36]. If the number of events is small, only a few exploratory variables can be investigated simultaneously [53]. In the present study there were only 28 cases of disease progressions or relapses (Table 2). This indicates that a maximum of two variables could be included in a multivariate Cox regression model.

In this study, the benefit of complete tumor resection and other treatment-related factors for progression-free survival was not investigated, because these variables were mostly unknown at the beginning of survival time (i.e., at diagnosis). To investigate a variable that is still elusive at the beginning of survival time or that changes over time, a time-dependent Cox regression must be used. For example, if the authors wish to know whether cancer patients’ cumulative dose of chemotherapy affects the length of time until the tumor progresses, they cannot stipulate the cumulative dose as a known quantity at the outset. Patients who survive longer will generally receive a higher total dose. However, this high cumulative dose is not the cause of longer disease control. To allow for this, the cumulative dose must be included in a Cox regression as a time-dependent variable. Time-dependent Cox regression is a procedure that requires particularly detailed information about the starting date of therapy, which is generally not provided by case series/reports extracted from literature. [14], [53].

### Limitations inherent to the concept of IPD meta-analyses

In addition, several limitations of this study are inherently in the concept of individual patient data (ITP) meta-analyses. First, there certainly was a selection bias, because the cases reported might have been published because of their rare or uncommon presentation and outcomes. Second, the data were gathered from different institutions during a relatively long period of time, and significant advances may have simultaneously occurred in diagnostic and therapeutic approaches. Last but not least, not all data assessed were available for every patient, which further restricts the number of variables assessable in a multivariate model [33].

## Conclusions

The clinical understanding of the three new members of the family of glioneuronal tumors - PGNT, RGNT and GNTNI - is currently in evolution. The present meta-analysis will hopefully contribute to a narrower diagnostic, therapeutic, and prognostic profile. However, the available data do not provide a solid basis to define the optimum treatment approach. It is proposed to establish a central register.

## Supporting Information

File S1 **Detailed description of references containing patient data.**
(DOC)

Checklist S1 **PRISMA 2009 Checklist.**
(DOC)
