# Supplementary material for: An Individual Patient Data Meta-Analysis on Characteristics and Outcome of Patients with Papillary Glioneuronal Tumor, Rosette Glioneuronal Tumor with Neuropil-Like Islands and Rosette Forming Glioneuronal Tumor of the Fourth Ventricle
Source: PLoS One. 2014 Jul 3;9(7):e101211. doi: 10.1371/journal.pone.0101211 (PMC4084640; doi:10.1371/journal.pone.0101211)
Supplement: File S1 — Detailed description of references containing patient data. (DOC) [file pone.0101211.s001.doc]

# **Additional file 1 – References**

1. Adachi, J.A; Nishikawa, R.; Hirose, T.; Matsutani, M. (2005): Mixed neuronal-glial tumor of the fourth ventricle and successful treatment of postoperative mutism with bromocriptine: case report. In: *Surgical Neurology* (63), pp. 375–379.
2. Adam, C.; Polivka, M.; Carpentier, A.; George, B.; Gray, F. (2007): Papillary glioneuronal tumor: not always a benign tumor? In: *Clinical Neuropathology* (26), pp. 119-124.
3. Agarwal, S.; Sharma, M.; Singh, G.; Suri, V.; Sarkar, C.; Garg, A. et al. (2012): Papillary glioneuronal tumor—a rare entity: report of four cases and brief review of literature. In: *Child's nervous system: ChNS : official journal of the International Society for Pediatric Neurosurgery* (28), pp. 1897–1904.
4. Agarwal, S.; Suri, V.; Rishi, A.; Shukla, B.; Garg, A.; Sharma, M.C et al. (2009): Glioneuronal tumor with neuropil-like islands: A new entity. In: *Neuropathology* (29), pp. 96–100.
5. Albanese, A.; Mangiola, A.; Pompucci, A.; Sabatino, G.; Gessi, M.; Lauriola, L.; Anile, C. (2005): Rosette-forming glioneuronal tumour of the fourth ventricle: report of a case with clinical and surgical implications. In: *Journal of Neuro-Oncology* (71), pp. 195–197.
6. Alturkustani, M.; Ang, L. (2012): Rosette-Forming Glioneuronal Tumour of the 4^th^ Ventricle in a NF1 Patient. In: Canadian Journal *of Neurological Sciences* (39), pp. 95-96.
7. Anan, M.; Inoue, R.; Ishii, K.; Abe, T.; Fujiki, M.; Kobayashi, H. et al. (2009): A rosette-forming glioneuronal tumor of the spinal cord: the first case of a rosette-forming glioneuronal tumor originating from the spinal cord. In: *Human Pathology* (40), pp. 898–901.
8. Arai, A.; Sasayama, T.; Tamaki, M.; Sakagami, Y.; Enoki, E.; Ohbayashi, C.; Kohmura, E. (2010): Rosette-Forming Glioneuronal Tumor of the Fourth Ventricle. Case Report. In: *Neurologia medico-chirurgica* (50), pp. 224–228.
9. Atri, S.; Sharma, M.; Sarkar, C.; Garg, A.; Suri, A. (2007): Papillary glioneuronal tumour: a report of a rare case and review of literature. In: *Child's Nervous System* (23), pp. 349–353.
10. Barbashina, V.; Salazar, P.; Ladanyi, M.; Rosenblum, M.; Edgar, M.A (2007): Glioneuronal Tumor With Neuropil-like Islands (GTNI): A Report of 8 Cases With Chromosome 1p/19q Deletion Analysis. In: *The American Journal of Surgical Pathology* (31), pp. 1196–1202.
11. Barnes, N.P; Pollock, J.R; Harding, B.; Hayward, R.D (2002): Papillary Glioneuronal Tumour in a 4-Year-Old. In: *Pediatric Neurosurgery* (36), pp. 266–270.
12. Benzagmout, M.; Karachi, C.; Mokhtari, K.; Capelle, L. (2013): Hemorrhagic papillary glioneuronal tumor mimicking cavernoma: Two case reports. In: *Clinical Neurology and Neurosurgery* (115 (2)), pp. 200–203.
13. Bisson, E.F; Pendlebury, W.W; Horgan, M.A (2005): Glioneuronal tumor with unique imaging and histologic features. Letter to the Editor. In: *Journal of Neuro-Oncology* (72), pp. 89–90.
14. Borges, G.; Bonilha, L.; Menezes, A.S; Souza Queiroz, L. de; Carelli, E.F; Zanardi, V.; Menezes, J.R (2004): Long term Follow-up in a Patient with Papillary Glioneuronal Tumor. In: *Arquivos de Neuro-Psiquiatria* (62 (3-B)), pp. 869–872.
15. Bouvier-Labit, C.; Daniel, L.; Dufour, H.; Grisoli, F.; Figarella-Branger, D. (2000): Papillary glioneuronal tumour: clinicopathological and biochemical study of one case with 7-year follow up. In: *Acta Neuropathologica* (99), pp. 321–326.
16. Bridge, J.A; Liu, X.; Sumegi, J.; Nelson, M.; Reyes, C.; Bruch, L.A et al. (2013): Identification of a Novel, Recurrent SLC44A1 PRKCA Fusion in Papillary Glioneuronal Tumor. In: *Brain pathology* (23 (2)), pp. 121–128.
17. Broholm, H.; Madsen, F.F.; Wagner, A.A.; Laursen, H. (2002): Papillary glioneuronal tumor – a new tumor entity. In: *Clinical Neuropathology* (21), pp. 1-4.
18. Buccoliero A.M.; Giordano, F.; Mussa, F.; Taddei, A.; Genitori, L.; Taddei, G.L (2006): Papillary glioneuronal tumor radiologically mimicking a cavernous hemangioma with hemorrhagic onset. In: *Neuropathology* (26), pp. 206–211.
19. Buccoliero, A.M.; Castiglione, F.; Degl'Innocenti, D.R.; Moncini, D.; Paglierani, M.; Sardi, I.; Giunti, L.; Giordano, F.; Sanzo, M.; Mussa, F.; Arico, M.; Genitori, L.; Taddei, G.L. (2012): Glioneuronal Tumor with Neuropil-Like Islands: Clinical, Morphologic, Immunohistochemical, and Molecular Features of Three Pediatric cases. In: *Pediatric and Developmental Pathology* (15), pp. 352-360.
20. Celli, P.; Caroli, E.; Giangaspero, F.; Ferrante, L. (2006): Papillary glioneuronal tumor. Case report and literature review. In: *Journal of Neuro-Oncology* (80), pp. 185–189.
21. Chakraborti, S.; Mahadevan, A.; Govindan, A.; Bhateja, A.; Dwarakanath, S.; Aravinda, H.R et al. (2012): Rosette-forming glioneuronal tumor — evidence of stem cell origin with biphenotypic differentiation. In: *Virchows Arch* (461), pp. 581–588.
22. Chen, L.; Piao, Y.; Xu, Q.; Yang, X.; Yang, H.; Lu, D. (2006): Papillary glioneuronal tumor: A clinicopathological and immunohistochemical study of two cases. In: *Neuropathology* (26), pp. 243–248.
23. Dim, D.C; Lingamfelter, D.C; Taboada, E. M.; Fiorella, R. M. (2006): Papillary glioneuronal tumor: a case report and review of the literature. In: *Human Pathology* (37), pp. 914–918.
24. Ebato, M.; Tsunoda, A.; Maruki, C.; Ikeya, F.; Okada M. (2003): Papillary glioneuronal tumor with highly degenerative pseudopapillary structure accompanied by specific abortive glial cells: a case report. In: *No Shinkei Geka* (31 (11)), pp. 1185-1190.
25. Ellezam, B.; Theeler, B.J.; Luthra, R.; Adesina, A. M.; Aldape, K.D.; Gilbert, M.R. (2012): Recurrent PIK3CA mutations in rosette-forming glioneuronal tumor. In: *Acta Neuropathologica* (123), pp. 285-287.
26. Epelbaum, S.; Kujas, M.; van Effenterre, R.; Poirier, J. (2006): Two cases of papillary glioneuronal tumours. In: *British Journal of Neurosurgery* (20 (2)), pp. 90–93.
27. Faria, C.; Miguéns, J.; Antunes, J.L; Barroso, C.; Pimentel, J.; do Carmo Martins, M. et al. (2008): Genetic alterations in a papillary glioneuronal tumor. In: *Journal of Neurosurgery: Pediatrics* (1), pp. 99–102.
28. Flannery, T.; Purce, A.; Harney, J.; McKinstry, S.; Ironside, J.W.; Herron, B. (2012): Bilateral non-contiguous atyplical papillary glioneuronal tumor: case report. In: *Clinical Neuropathology* (31), pp.77-80.
29. Fraum, T.J; Barak, S.; Pack, S.; Lonser, R.R. Fine H.A; Quezado, M.; Iwamoto, F.M (2012): Spinal cord glioneuronal tumor with neuropil-like islands with 1p/19q deletion in an adult with low-grade cerebral oligodendroglioma. In: *Journal of Neuro-Oncology* (107), pp. 421–426.
30. Frydenberg, E.; Laherty, R.; Rodriguez, M.; Ow-Yang, M.; Steel, T. (2010): A rosette-forming glioneuronal tumour of the pineal gland. In: *Journal of Clinical Neuroscience* (17), pp. 1326–1328.
31. Fushimi, Y.; Miyasaki, A.; Taki, H.; Aoyama, K.; Hirato, J.; Kanagaki, M.; Togashi, K. (2011): Rosette-forming glioneuronal tumor of the ventricle with bilateral olivary degeneration. In: *Japanese Journal of Radiology* (29), pp. 445-448.
32. Gelpi, E.; Preusser, M.; Czech, T.; Slavc, I.; Prayer, D.; Budka, H. (2007): Papillary glioneuronal tumor. In: *Neuropathology* (27), pp. 468–473.
33. Gessi, M.; Lambert, S.R; Lauriola, L.; Waha, A.; Collins, V.P; Pietsch, T. (2012): Absence of KIAA1549-BRAF fusion in rosette-forming glioneuronal tumors of the fourth ventricle (RGNT). In: *Journal of Neuro-Oncology* (110), pp. 21–25.
34. Gessi, M.; Waha, A.; Setty, P.; Waha, A.; Pietsch, T. (2011): Analysis of KIAA1549-BRAF fusion status in a case of rosette-forming glioneuronal tumor of the fourth ventricle (RGNT). In: *Neuropathology* (31), pp. 654–657.
35. Ghosal, N.; Furtado, S.V; Hegde, A.S (2010): Rosette Forming Glioneuronal Tumor Pineal Gland and Tectum: An Intraoperative Diagnosis on Smear Preparation. In: *Diagnostic Histopathology* (38), pp. 590–593.
36. Govindan, A.; Mahadevan, A.; Bhat, D.I; Arivazhagan, A.; Chakraborti, S.; Suja, M.S et al. (2009): Papillary glioneuronal tumor—evidence of stem cell origin with biphenotypic differentiation. In: *Journal of Neuro-Oncology* (95), pp. 71–80.
37. Guo, S.P.; Zhang, F.; Li, Q.L., Li, Q.; Wang, W.L.; Li, F.F. (2008): Papillary glioneuronal tumor – contribution to a new tumor entity and literature review. In: *Clinical Neuropathology* (27), pp. 72-77.
38. Harris, B.T; Horoupian, D.S (2000): Spinal cord glioneuronal tumor with “rosetted” neuropil islands and meningeal dissemination: a case report. In: *Acta Neuropathologica* (100), pp. 575–579.
39. Hsu, C.; Kwan, G.; Lau, Q.; Bhuta, S. (2012): Rosette-forming glioneuronal tumour: Imaging features, histopathological correlation and a comprehensive review of literature. In: *British Journal of Neurosurgery* (26 (5)), pp. 668–673.
40. Husain, N.; Husain, M. (2009): Endoscopic diagnosis of a pineal papillary glioneuronal tumor with extensive ventricular involvement: Case report with review of literature. In: *Neurology India* (57 (6)), pp. 792–795.
41. Ishizawa, T.; Komori, T.; Shibahara, J.; Ishizawa, K.; Adachi, J.; Nishikawa, R. et al. (2006): Papillary glioneuronal tumor with minigemistocytic components and increased proliferative activity. In: *Human Pathology* (37), pp. 627–630.
42. Iżycka-Świeszewska, E.; Majewska, H.; Szurowska, E.; Mazurkiewicz-Bełdzińska, M.; Drożyńska, E. (2008): Papillary glioneuronal tumour of the precentral gyrus. In: *Folia Neuropathologica* (46 (2)), pp. 158–163.
43. Jacques, T.S; Eldridge, C.; Patelt, A.; Saleem, N.M; Powell, M.; Kitchen, N.D et al. (2005): Mixed glioneuronal tumour of the fourth ventricle with prominent rosette formation. In: *Neuropathology and Applied Neurobiology* (32), pp. 217–220.
44. Javahery, R.J; Davidson, L.; Fangusaro, J.; Finlay, J.L; Gonzalez-Gomez, I.; McComb, J.G (2009): Aggressive variant of a papillary glioneuronal tumor. Report of 2 cases. In: *Journal of Neurosurgery: Pediatrics* (3), pp. 46–52.
45. Johannesma, P.C; van der Klift, H.M; van Grieken, N.C.T; Troost, D.; te Riele, H.; Jacobs, M.A.J.M et al. (2011): Childhood brain tumours due to germline bi-allelic mismatch repair gene mutations. In: *Clinical Genetics* (80), pp. 243–255.
46. Johnson, M.; Pace, J.; Burroughs, J.F (2006): Fourth ventricle rosette-forming glioneuronal tumor. In: *Journal of Neurosurgery: Pediatrics* (105), pp. 129–131.
47. Joseph, V.; Wells, A.; Kuo, Y.; Halcrow, S.; Brophy, B.; Scott, G. et al. (2009): The ‘rosette-forming glioneuronal tumor’ of the fourth ventricle. In: *Neuropathology* (29), pp. 309–314.
48. Karafin, M.; Jallo, G.I.; Ayars, M.; Eberhart, C.G.; Rodriguez, F.J. (2011): Rosette forming glioneuronal tumor in association with Noonan syndrome: pathobiological implications. In: *Clinical Neuropathology* (30), pp. 297-300.
49. Kemp, S.; Achan, A.; Ng, T.; Dexter, M.A.J (2012): Rosette-forming glioneuronal tumour of the lateral ventricle in a patient with neurofibromatosis 1. In: *Journal of Clinical Neuroscience* (19), pp. 1180–1181.
50. Keyvani, K.; Rickert, C.H; Wild, K. von; Paulus, W. (2001): Rosetted glioneuronal tumor: a case with proliferating neuronal nodules. In: *Acta Neuropathologica* (101), pp. 525–528.
51. Kinno, M.; Ishizawa, K.; Shimada, S.; Masaoka, H.; Doi, M.; Seyama, S. et al. (2010): Cytology is a useful tool for the diagnosis of rosette-forming glioneuronal tumour of the fourth ventricle: a report of two cases. In: *Cytopathology* (21), pp. 194–197.
52. Komori, T.; Scheithauer, B.W; Anthony, D.; Rosenblum, M.; McLendon, R.; Scott, R. et al. (1998): Papillary Glioneuronal Tumor: A New Variant of Mixed Neuronal-Glial Neoplasm. In: *The American Journal of Surgical Pathology* (22 (10)), pp. 1171–1183.
53. Komori, T.; Scheithauer, B.W; Hirose, T. (2002): A Rosette-Forming Glioneuronal Tumor of the Fourth Ventricle. Infratentorial Form of Dysembryoplastic Neuroepithelial Tumor? In: *The American Journal of Surgical Pathology* (26 (5)), pp. 582–591.
54. Lamszus, K.; Makrigeorgi-Butera, M.; Laas, R.; Westphal, M.; Stavrou, D. (2003): September 2002: 24-year-old female with a 6-month history of seizures. In: *Brain pathology* (1), pp. 115–117.
55. Lavrnic, S.; Macvanski, M.; Ristic-Balos, D.; Gavrilov, M.; Damjanovic, D.; Gavrilovic, S. et al. (2012): Papillary Glioneuronal Tumor: Unexplored Entity. In: *Journal of neurological surgery. Part A, Central European neurosurgery* (73), pp. 224–229.
56. Li, Y.M; Li, W.Q; Pan, Y.; Lu, Y.C; Long, N.Y; Tao, X.F; Yu, H.Y (2009): Rosette-forming Glioneuronal Tumour of the Fourth Ventricle with Previous Intratumoural Haemorrhage: Case Report and Review of the Literature. In: *The Journal of International Medical Research* (37), pp. 958–966.
57. Lu, J.; Scheithauer, B.W; Sharma, P.; Scott, J.N; Parney, I.F; Hader, W. et al. (2009): Multifocal complex glioneuronal tumor in an elderly man: an autopsy study: Case Report. In: *Neurosurgery* (64(6)), pp. 1193–1195.
58. Luan, S.H; Zhuang, D.X; Sun, L.L; Huang, F. (2010): Rosette-forming glioneuronal tumor (RGNT) of the fourth ventricle: Case report and review of literature. In: *Clinical Neurology and Neurosurgery* (112), pp. 362–364.
59. Mahajan, H.; Varikatt, W.; Dexter, M.; Boadle, R.; Ng, T. (2010): Papillary glioneuronal tumor of the frontal lobe. In: *Journal of Clinical Neuroscience* (17), pp. 534–536.
60. Marhold, F.; Preusser, M.; Dietrich, W.; Prayer, D.; Czech, T. (2008): Clinicoradiological features of rosette-forming glioneuronal tumor (RGNT) of the fourth ventricle: report of four cases and literature review. In: *Journal of Neuro-Oncology* (90), pp. 301–308.
61. Matsko, D.E. (2008): Papillary glioneuronal tumor is a new nosological entity in the WHO classification of central nervous system tumors. In: *Arkhiv patologii* (70), pp. 45-46.
62. Matyja, E.; Grajkowska, W.; Nauman, P.; Ozieblo, A.; Bonicki, W. (2011): Rosette-forming glioneuronal tumor of the fourth ventricle with advanced microvascular proliferation – a case report. In: *Neuropathology* (31), pp. 427–432.
63. Min, H.S; Lee, S.H; Yoo, H.; Myung, J.K; Hong, E.K; Park, S. (2010): Cytogenetic study of glioneuronal tumor with neuropil-like islands: A case report. Case Report. In: *Neuropathology* (30), pp. 420–426.
64. Myung, J.K; Byeon, S.; Kim, B.; Suh, J.; Kim, S.; Park, C. et al. (2011): Papillary Glioneuronal Tumors: A Review of Clinicopathologic and Molecular Genetic Studies. In: *The American Journal of Surgical Pathology* (35), pp. 1794–1805.
65. Newton, H.B.; Dalton, J.; Ray-Chaudhury, A.; Gahbauer, R.; McGregor, J. (2008): Aggressive papillary glioneuronal tumor: case report and literature review. In: *Clinical Neuropathology* (27), pp. 317-324.
66. Phi, J.H; Park, S.; Chae, J.H; Wang, K.; Cho, B.; Kim, S. (2010): Papillary Glioneuronal Tumor Present in a Patient With Encephalocraniocutaneous Lipomatosis: Case Report. In: *Neurosurgery* (67 (4)), pp. 1165–1169.
67. Pimentel, J.; Resende, M.; Vaz, A.; Reis, A.M; Campos, A.; Carvalho, H.; Honavar, M. (2008): Rosette-forming Glioneuronal Tumor: Pathology Case Report. In: *Neurosurgery* (62 (5)), pp. 1162–1163.
68. Pimentel, J.; Barroso, C.; Miguéns, J.; Firmo, C.; Antunes, J.L. (2009): Papillary glioneuronal tumor – Prognostic value of the extension of surgical resection. In: *Clinical Neuropathology* (28), pp. 287-294.
69. Podlesek, D.; Geiger, K.; Hendry, D.J; Schackert, G.; Krex, D. (2011): Rosette-forming glioneuronal tumor of the fourth ventricle in an elderly patient. In: *Journal of Neuro-Oncology* (103), pp. 727–731.
70. Poliani, P.L; Sperli, D.; Valentini, S.; Armentano, A.; Bercich, L.; Bonetti, M.F et al. (2009): Spinal glioneuronal tumor with neuropil-like islands and meningeal dissemination: histopathological and radiological study of a pediatric case. In: *Neuropathology* (20), pp. 574–578.
71. Prayson, R.A (2000): Papillary Glioneuronal Tumor. In: *Archives of Pathology & Laboratory Medicine* (124), pp. 1820–1823.
72. Prayson, R.A; Abramovich, C.M (2000): Glioneuronal Tumor with Neuropil-like Islands. In: *Human Pathology* (31 (11)), pp. 1435–1437.
73. Preusser, M.; Dietrich, W.; Czech, T.; Prayer, D.; Budka, H.; Hainfellner, J.A. (2003): Rosette-forming glioneuronal tumor of the fourth ventricle. In: *Acta Neuropathologica* (106), pp. 506-508.
74. Radotra, B.D; Kumar, Y.; Bhatia, A.; Mohindra, S. (2007): Papillary glioneuronal tumor: a new entity awaiting inclusion in WHO classification. In: *Diagnostic Pathology* (2:6), pp. 1–5.
75. Rainov, N.G.; Wagner, T.; Heidecke, V. (2010): Rosette-Forming Glioneuronal Tumor of the Fourth Ventricle. In: *Central European Neurosurgery* (71), pp. 219-221.
76. Rickert, C.H.; Jasper, M.; Sepehrnia, A.; Jeibmann, A. (2006): Rosetted glioneuronal tumour of the spine: clinical, histological and cytogenetic data. In: *Acta Neuropathologica* (112), pp. 231-233.
77. Ruppert, B.; Welsh, C.T; Hannah, J.; Giglio, P.; Rumboldt, Z.; Johnson, I. et al. (2011): Glioneuronal tumor with neuropil-like islands of the spinal cord with diffuse leptomeningeal neuraxis dissemination. In: *Journal of Neuro-Oncology* (104), pp. 529–533.
78. Scheithauer, B.W; Silva, A.I; Ketterling, R.P; Pula, J.H; Lininger, J.F; Krinock, M.J (2009): Rosette-forming glioneuronal tumor: Report of a Chiasmal-optic nerve example in Neuro-fibromatosis Type 1: Special Pathology Report. In: *Neurosurgery* (64 (4)), pp. 771–772.
79. Scholz, M.; Hoischen, A.; Radlwimmer, B.; Weber, R.; Harders, A.; Reifenberger, G.; Riemenschneider, M.J. (2009): Rosetted glioneuronal tumor of the spine with overtly anaplastic histological features. In: *Acta Neuropathologica* (117), pp. 591-593.
80. Shah, M.N; Leonard, J.R; Perry, A. (2010): Rosette-forming glioneuronal tumors of the posterior fossa. Report of 6 cases. In: *Journal of Neurosurgery: Pediatrics* (5), pp. 98–103.
81. Sharma, P.; Swain, M.; Padua, M. de; Ranjan, A.; Lath, R. (2011): Rosette-forming glioneuronal tumors: A report of two cases. In: *Neurology India* (59 (2)), pp. 276–280.
82. Solis, O.E; Mehta, R.I; Lai, A.; Mehta, R.I; Farchoukh, L.O; Green, R.M et al. (2011): Rosette-forming glioneuronal tumor: a pineal region case with IDH1 and IDH2 mutation analyses and literature review of 43 cases. In: *Journal of Neuro-Oncology* (102), pp. 477–484.
83. Tan, C.C; Gonzales, M.; Veitch, A. (2008): Clinical Implications of the infratentorial Rosette-forming glioneuronal tumor: Case Report. In: *Neurosurgery* (63 (1)), pp. 175–176.
84. Tanaka, Y.; Yokoo, H.; Komori, T.; Makita, Y.; Ishizawa, T.; Hirose, T.; Ebato, M.; Shibahara, J.; Tsukayama, C.; Shibuya, M.; Nakazato, Y. (2005): A distinct pattern of Olig2-positive cellular distribution in papillary glioneuronal tumors: a manifestation of the oligodendroglial phenotype? In: *Acta Neuropathologica* (110), pp. 39-47.
85. Teo, J.; Gultekin, S.H. Bilsky M.; Gutin, P.; Rosenblum, M. (1999): A Distinctive Glioneuronal Tumor of the Adult Cerebrum With Neuropil-Like (Including "Rosetted") Islands: Report of 4 Cases. In: *The American Journal of Surgical Pathology* (23 (5)), pp. 502–510.
86. Tsukayama, C.; Arakawa, Y. (2002): A papillary glioneuronal tumor arising in an elderly woman: a case report. In: *Brain Tumor Pathology* (19), pp. 35–39.
87. Ulivieri, S.; Oliveri, G.; Cerase, A.; Miracco, C. (2010): Papillary glioneuronal tumor of the fourth ventricle: case report and review of the literature. In: *Il Giornale di chirurgia* (31 (8/9)), pp. 368–370.
88. Vajtai, I.; Arnold, M.; Kappeler, A.; Jeless, O.; Lukes, A.; Mariani, L.; Paulus, W. (2007): Rosette-forming glioneuronal tumor of the fourth ventricle: Report of two cases with a differential diagnostic overview. In: *Pathology - Research and Practice* (203), pp. 613-319.
89. Vajtai, I.; Kappeler, A.; Lukes, A.; Arnold, M.; Ridolfi Lüthy, A.; Leibundgut, K. (2006): Papillary glioneuronal tumor. In: *Pathology - Research and Practice* (202), pp. 107–112.
90. Wang, Y.; Xiong, J.; Chu, S.; Liu, Y.; Cheng, H.; Wang, Y., Zhao, Y.; Mao, Y. (2009): Rosette-forming glioneuronal tumor: report of an unusual case with intraventricular dissemination. In: *Acta Neuropathologica* (118), pp. 813-819.
91. Williams, S.R; Joos, B.W; Parker, J.C; Parker, J.R (2008): Papillary Glioneuronal Tumor: A Case Report and Review of the Literature. In: *Annals of Clinical & Laboratory Science* (38 (3)), pp. 287–292.
92. Xiao, H.; Ma, L.; Lou, X.; Gui, Q. (2011): Papillary Glioneuronal Tumor: Radiological Evidence of a Newly Established Tumor Entity. In: *Journal of Neuroimaging* (21), pp. 297–302.
93. Xiong, J.; Liu, Y.; Chu, S.; Chen, H.; Chen, H.X; Mao, Y.; Wang, Y. (2012): Rosette-forming glioneuronal tumor of the septum pellucidum with extension to the supratentorial ventricles: Rare case with genetic analysis. In: *Neuropathology* (32), pp. 301–305.
94. Yin, B.; Liu, L.; Chen, X.; Li, K.; Geng, D. (2012): Rosette-forming glioneuronal tumor of the fourth ventricle. In: *Journal of Neuroradiology: Organe officiell de la Société Fran*ç*aise de Neuroradiologie* (39 (2)), pp. 129-130.
95. Zhong, D.; Zhao, Y.; Gao, J.; Zeng, X.; Ma, W. (2010): Special morphological papillary glioneuronal tumor. In: *Chinese Journal of Contemporary Neurology and Neurosurgery* (10), pp. 381-386.
